# Dedifferentiated liposarcoma primary to the chest wall with spontaneous shrinking: report of a case

**DOI:** 10.1186/s40792-023-01606-x

**Published:** 2023-02-15

**Authors:** Yuki Itagaki, Akira Fukunaga, Hironobu Takano, Kazuyuki Yamamoto, Kohei Nishigami, Tatsunosuke Ichimura, Hiroto Manase, Masahiko Obata, Tatsuya Kato, Satoshi Hirano

**Affiliations:** 1 Department of Surgery, Japanese Red Cross Asahikawa Hospital, 1-1, Akebono 1-1, Hokkaido, Asahikawa, 070-8530 Japan; 2 Department of Gastroenterological Surgery II, Faculty of Medicine, Hokkaido University, North 15 West 7, Kita-Ku, Hokkaido, Sapporo, 060-8638 Japan; 3 Department of Thoracic Surgery, Japanese Red Cross Asahikawa Hospital, 1-1, Akebono 1Joh, 1Choume, Hokkaido, Asahikawa, , 070-8530 Japan; 4 Department of Surgical Pathology, Japanese Red Cross Asahikawa Hospital, 1-1, Akebono 1Joh, 1Choume, Hokkaido, Asahikawa, 070-8530 Japan; 5 Department of Thoracic Surgery, Hokkaido University Hospital, North 14 West 5, Kita-Ku, Hokkaido, Sapporo, 060-8648 Japan

## Abstract

An 80-year-old man presented to our emergency department complaining of a mass on the right side of his chest and pain in the right flank of his back. A chest computed tomography (CT) scan showed a relatively heterogenous oval-shaped tumor measuring 7.5 × 6.0 cm eroded to the 8th rib, with slightly dense fluid accumulation inside and calcification of the tumor wall. A 1-month follow-up CT scan showed spontaneous shrinkage of the tumor. The tumor was completely excised from the thoracic wall and the wall was reconstructed with a polytetrafluoroethylene mesh. Pathological examination showed coagulation necrosis in the chest wall tumor, but immunohistochemical staining revealed murine double minute 2- and Cyclin-dependent kinase 4-positive cells with irregular nuclear size and bizarre morphology. Therefore, dedifferentiated liposarcoma (DDLPS) was the final pathological diagnosis. Remarkable infiltration of CD8+ lymphocytes into the tumor was observed, along with a 90% positive ratio for programmed cell death-ligand 1. The patient has been followed-up for 1 year without any recurrence, despite not receiving any additional treatment. Liposarcoma is one of the most common types of soft tissue sarcomas; however, spontaneous regression of primary DDLPS arising from the chest wall is extremely rare. Herein, we report a case of DDLPS primary to the chest wall with spontaneous regression, probably due to a spontaneously induced T cell response.

## Introduction

Liposarcoma is one of the most common types of soft tissue sarcoma, occurring in the retroperitoneum in 50% of cases and the extremities in 25% of cases [1]. Primary liposarcoma of the chest wall is extremely rare [2]. Complete surgical resection is considered to be the most effective therapy [1]. Although various types of tumors with spontaneous regression have been reported, such as hepatocellular carcinoma, renal cell carcinoma, melanoma, and neuroblastoma, the rate of spontaneous regression is less than 0.001% [3–5]. Herein, we report a case of dedifferentiated liposarcoma (DDLPS) arising from the chest wall with spontaneous shrinkage, which revealed remarkable tumor infiltration of CD8+ lymphocytes and a 90% positive ratio of programmed cell death-ligand 1 (PD-L1), suggesting a spontaneously-induced T cell response.

## Case report

**Fig. 1:**
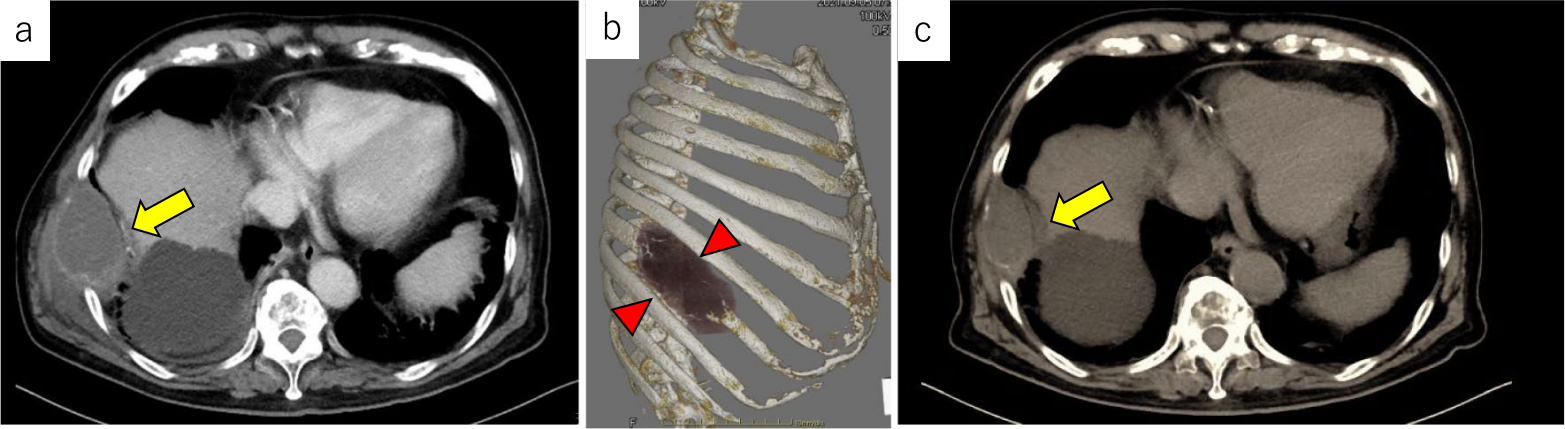
CT scan images of the chest wall tumor. **a** Axial CT image at the first visit. **b** 3D-reconstructed CT image of the tumor. **c** CT scan image of the tumor with spontaneously regression after 1 month

**Fig. 2:**
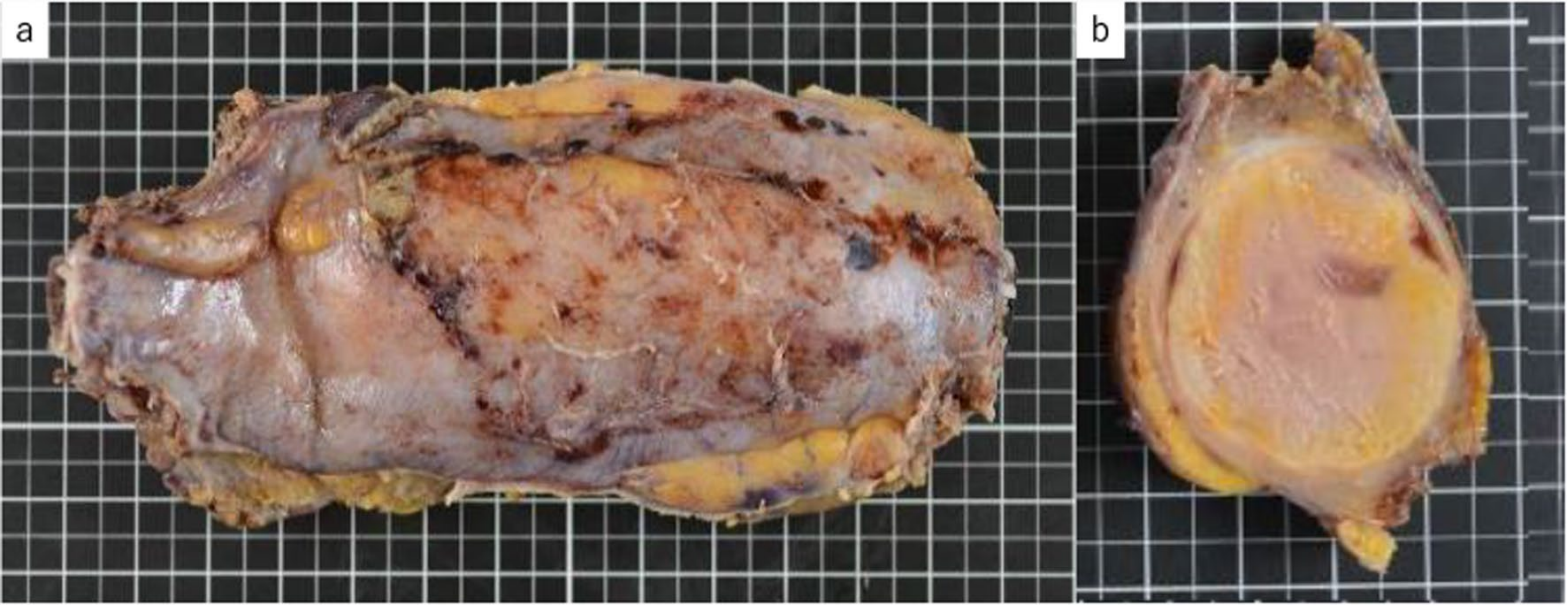
Gross appearance of the resected specimen. **a** Gross appearance resected specimen combined with 7th–9th ribs and intercostal muscle resection after fixation. **b** Cut surface showed bimorphic of gray-white and pale-yellow components with a heterogeneous appearance

**Fig. 3:**
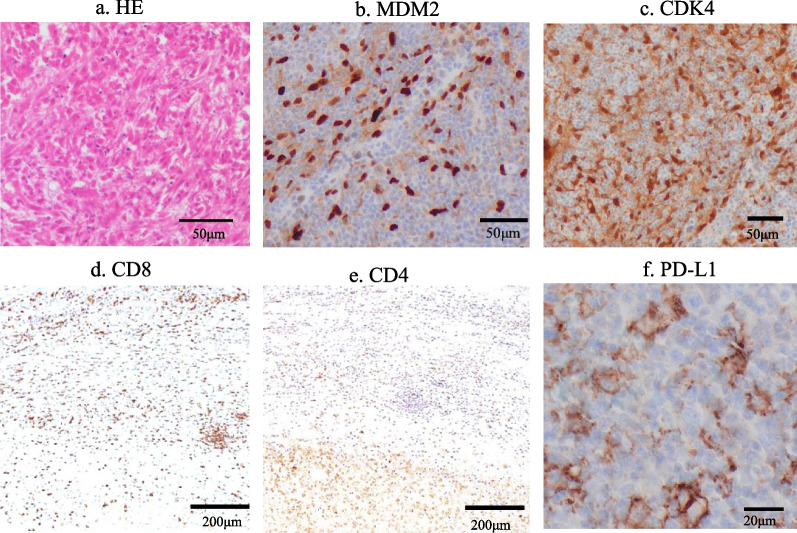
Microscopic and immunohistochemical findings. **a** HE-staining showing dedifferentiated liposarcoma with necrotic foci. **b**, **c** Tumor cells around necrotic tissue showing high MDM2 and CDK4-expression in the resected margin. **d**, **e** CD8 + and CD4 + T cell infiltration was detected by immunostaining. **f** 90% of tumor cells were positive for PD-L1 by immunostaining

A 80-year-old man came to our emergency department complaining of a mass in the right side of his chest and the pain of the right flank of his back. The gastrointestinal symptoms such as vomiting, diarrhea followed by the first pain 2 months before the first visit. The patient’s medical history included hypertension, dyslipidemia, combined pulmonary fibrosis and emphysema, and cerebral infarction. He was a heavy ex-smoker with a 45 pack-year. Chest computed tomography (CT) scan showed a low-density, relatively heterogenous, chest wall tumor measuring 75 × 60 × 55 mm, eroded to the 8th ribs, with slightly dense fluid accumulation inside and calcification of the tumor wall (Fig. 1a and b). Diaphragmatic invasion was also suspected. Although needle biopsy revealed a few sarcomatoid cells with large chromatin-rich nuclei and lymphocytic infiltration, suggesting the possibility of sarcoma, a pathological diagnosis could not be confirmed. The patient was referred to the department of thoracic surgery for treatment. A 1-month follow-up CT showed spontaneous regression of the tumor (70 × 57 × 34 mm) (Fig. 1c). Although the tumor had spontaneously regressed and a benign disorder such as infection was suspected, a complete surgical resection of the tumor and chest wall was planned, because the possibility of malignant disease remained. Thoracoscopic examination revealed that the tumor remained in the subpleural layer without reaching the thoracic cavity. We decided to excise the tumor along with the 7th–9th ribs and the connected intercostal muscles (Fig. 2a). We performed excision of the tumor along with the 8th rib and the connected intercostal muscles. The posterior margin was 1 cm and anterior margin was 2 cm, which was macroscopically appeared far enough away from the tumor. The resected thoracic wall was reconstructed with a 1-mm-thick polytetrafluoroethylene mesh. Macroscopically, the cut surface of the resected specimen was bimorphic, being both gray-white and pale yellow (Fig. 2b). Pathologically, the chest wall tumor showed necrotic foci and almost no viable cells (Fig. 3a); however, immunohistochemical staining revealed murine double minute 2 (MDM2) and cyclin-dependent kinase 4 (CDK4)-positive nuclear cells with irregularities in nuclear size and bizarre nuclear morphology, as shown in Figs. 3b and c. Therefore, DDLPS was the final pathological diagnosis. The ribs had been destroyed by inflammatory cell infiltration, including foamy macrophages. Immunohistochemical staining was also performed on the posterior rib stump, and MDM2/CDK4-positive cells were found buried in lymphocyte clusters within the pericostal soft tissue, indicating a positive margin. The tumor showed a large amount of CD8+ and CD4+ T cell infiltration around the tumor (Figs. 3d and e), and 90% of tumor cells were positive for PD-L1 (Fig. 3f). The patient had no postoperative events and was discharged on the eighth postoperative day. The patient has been followed-up for 1 year without any recurrence, despite not receiving any additional treatment.

## Discussion

Herein, we report a case of spontaneous regression of a DDLPS arising from the chest wall without any treatment. The histological types of liposarcoma are currently classified by the WHO as well-differentiated, myxoid, round cell, round cell, pleomorphic, and DDLPS [6]. Histological type is an important prognostic factor for liposarcoma, and DDLPS has the highest local recurrence rate (41–52%) [7]. The 5-year survival rate is reported to be 57.2% for DDLPS, and 95.5% for well-differentiated liposarcoma [2]. DDLPS occurs in late adult life with no sexual differences. More than 80% of DDLPS cases occur in the retroperitoneum and extremities; occurrences in the spermatic cord and other sites such as the chest wall or internal trunk are extraordinary rare [8]. To our knowledge, this is the first report of spontaneous regression of DDLPS primarily situated in the chest wall.

Spontaneous regression of tumors is rare but has been well-documented [3, 5]. As thinking about the triggers of spontaneous regression of the tumor, we only performed needle biopsy. We also did not administer any chemotherapy nor drugs. Furthermore, the patient complained of the vomiting and diarrhea before the right-side abdominal pain. However, there have been no supportive evidence or report which suggests the relation between biopsy or gastroenterological symptoms and the spontaneous shrinkage of the tumor. Recently, some studies have reported the relationships between spontaneous regression and programmed cell death 1 (PD-1) positive tumor-infiltrating lymphocytes (TIL) and PD-L1 positive tumor cells. Spontaneous regression of hepatocellular carcinoma has been reported, in cases where CD8+ TIL and PD-L1 positive cancer cells were detected [3]. Similar findings were observed in the present case. The tumor showed marked necrosis, many CD8+ T cell lymphocytes infiltrated the tumor wall, and the tumor cells showed high rates of PD-L1 expression. As CD8+ T cells are major killers of neoplastic cells [3, 9], we speculated that CD8+ T cells attack tumor cells that express PD-L1. As tumor necrosis and infiltrating lymphocytes existed simultaneously in the present case, we also speculate that host immunity promoted spontaneous regression and necrosis of the tumor. The first therapeutic choice for liposarcoma is complete resection, as in the present case [1]. Positive histological margins generally require additional treatment. However, no local recurrence has been observed in this case where treatment was not provided, despite a positive histological margin. Thus, it is possible that tumor immunity continues to function after surgery.

The presence of tumor-infiltrating lymphocytes and PD-L1 expression are both generally promoted by anti PD-1/L1 blockade [10]. However, Zhang et al. found no significant difference in metastasis-free or overall survival in cases of liposarcoma, regardless of PD-L1 expression [11]. The effectiveness of immune checkpoint blockade therapy has recently been investigated in an open-label phase II study of anti-PD-1 monotherapy in patients with histological evidence of metastatic or surgically unresectable locally advanced sarcoma. Pembrolizumab demonstrated a 40% overall response rate in DDLPS [12]. Furthermore, a phase II study of neoadjuvant checkpoint blockade in patients with resectable DDLPS is in progress [13]. Cases with spontaneous regression of the tumor, such as the present case, may receive the most benefit from the anti PD-1/L1 blockade. Further studies may lead to more practical therapeutic options for liposarcomas.

## Conclusions

Here, we report a case of DDLPS originating from the chest wall with spontaneous regression. The tumor was infiltrated by CD8+ T cells and high rates of PD-L1 expression, suggesting a spontaneously induced T cell response.

## Data Availability

Not applicable.

## Abbreviations

DDLPS: Dedifferentiated liposarcoma
CT: Computed tomography
PD-1: Programmed cell death 1
PD-L1: Programmed cell death ligand 1
TIL: Tumor-infiltrating lymphocytes
MDM2: Murine double minute 2
CDK4: Cyclin-dependent kinase 4
